# A review of recent developments in rare earth-doped nanophosphors for emerging technological applications

**DOI:** 10.1039/d5ra03126e

**Published:** 2025-06-12

**Authors:** R. Kiran, Nagaraj Kamath, M. I. Sayyed, Aljawhara H. Almuqrin, Sudha D. Kamath

**Affiliations:** a Department of Physics, Manipal Institute of Technology, Manipal Academy of Higher Education Manipal Karnataka India sudha.kamath@manipal.edu; b Department of Humanities and Management, Manipal Institute of Technology, Manipal Academy of Higher Education Manipal Karnataka India; c Renewable Energy and Environmental Technology Center, University of Tabuk Tabuk 47913 Saudi Arabia; d Department of Physics, College of Science, Princess Nourah bint Abdulrahman University P.O. Box 84428 Riyadh 11671 Saudi Arabia

## Abstract

This article explores the synthesis of rare-earth-doped nanophosphors (RENPs), focusing on selecting optimal host materials like tungstates, vanadates, and aluminates to enhance luminescent properties. The incorporation of rare-earth elements, such as Eu^3+^, Tb^3+^, Dy^3+^, Sm^3+^, *etc.*, significantly improves emission spectra and efficiency by utilizing their unique electronic configurations and interactions with the host lattice. Nanophosphors were synthesized through sol–gel, combustion, and microwave-assisted methods, resulting in materials with tunable photoluminescence, high luminous efficiency, and appropriate chromaticity for wide-bandgap lighting applications. The study further investigates upconversion nanophosphors (UCNPs), demonstrating exceptional attributes like long luminescence lifetimes, high sensitivity, low energy loss, and tunable emission spectra. These characteristics make UCNPs ideal for advanced sensing applications. Additionally, RENPs exhibit great potential in thermoluminescence dosimetry, providing accurate radiation dose measurements with minimal fading and energy-dependent responses, thus advancing both medical and environmental dosimetry.

## Abstract 2

This article explores the synthesis of rare-earth-doped nanophosphors (RENPs), focusing on selecting optimal host materials like tungstates, vanadates, and aluminates to enhance luminescent properties.

## Graphical abstract

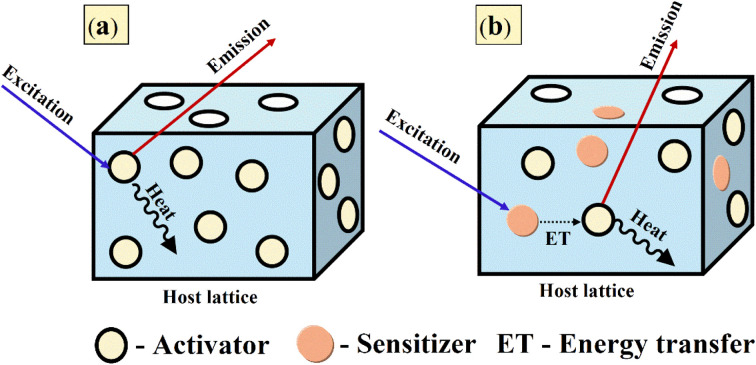

## 1. Introduction

**Fig. 1:**
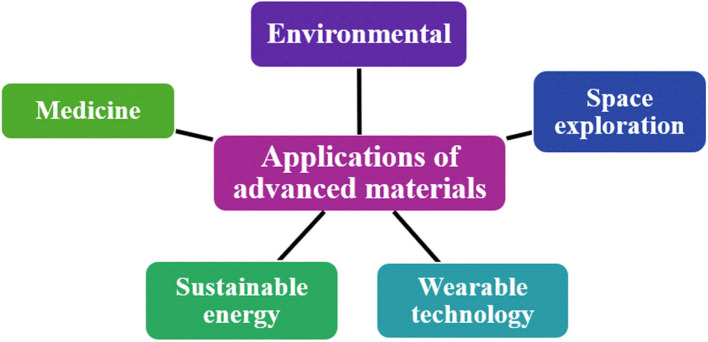
Various applications of advanced materials.

The domain of materials science and engineering is progressing at an accelerated pace, driven by the growing demand for materials that can meet the evolving needs of modern technology across diverse industrial, scientific, and commercial sectors.^1–4^ In material design, a key consideration is not only the material's versatility across various applications but also its ability to overcome the challenges encountered in real-world implementation.^5–7^ As a result, extensive global research is focused on developing novel materials that satisfy the rigorous demands of both everyday and cutting-edge technological applications.^8^Fig. 1 displays the various applications of advanced materials in modern science.^9^

**Fig. 2:**
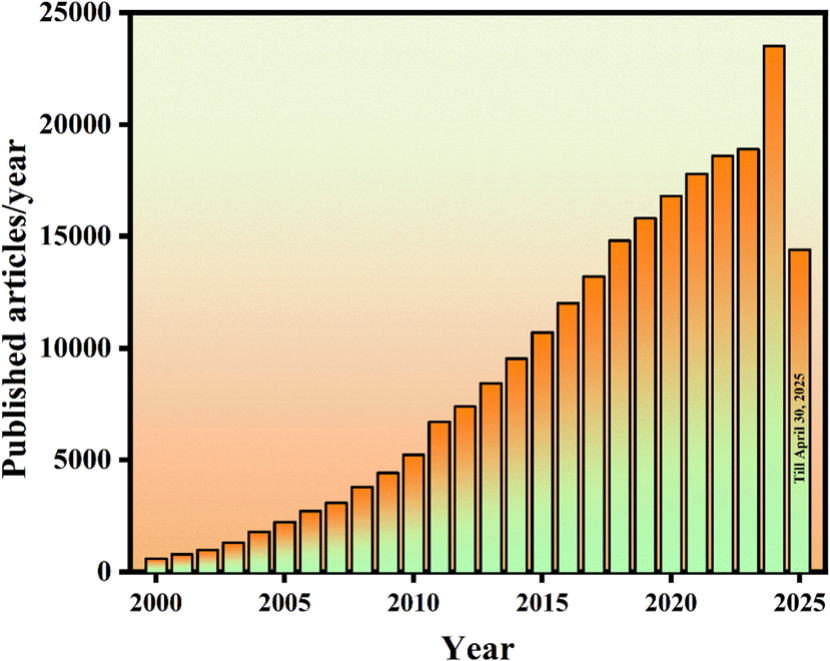
Publications per year with the keyword “nano phosphor” (Google Scholar, accessed April 30, 2025).

Rare earth-doped phosphors are one such unique class of materials that have attracted considerable attention in materials science due to their exceptional ability to emit controlled light, alongside remarkable thermal and chemical stability.^10^ Over time, a wide variety of host materials for phosphors have been explored in the literature, each contributing distinct properties that enhance the performance and applicability of phosphor-based systems.^11^ The continued exploration of novel host matrices remains critical for advancing the efficiency and functionality of phosphors, particularly for their application in an expanding range of technological fields. In recent years, the integration of nanotechnology with phosphors has led to the synthesis of rare earth-doped nanophosphors (RENPs), which have quickly emerged as a potential candidate to enhance the performance of light-emitting diodes (LEDs).^12^ A nanophosphor (NP) is a luminescent material with a particle size typically ranging from 1 to 100 nanometers. Fig. 2 illustrates the volume of research articles published over the past 24 years within the domain of NPs.

It is important to note that the unique spectroscopic properties of RENPs arise not from the quantum size effects but from a combination of factors, including particle size, doping composition, site symmetry, dopant-ligand distance, crystalline phase, and the strength of coordination. Surface effects also play a significant role in determining their overall luminescent behaviour.^13^ Furthermore, the reduction of phosphor particle size to the nanoscale confers significant advantages, including superior optical, thermal, and mechanical properties. These enhanced characteristics make NPs central to the development of next-generation, energy-efficient lighting systems. For example, the quantum efficiency (QE) of LaSr_2_AlO_5_:1 mol% Sm^3+^ nanophosphor was measured at 94.49%, making it comparable to commercially available phosphors for LED applications.^14^ Additionally, the luminescent lifetime of the NPs spans a wide range, from a few microseconds and milliseconds to several seconds and even minutes, highlighting their potential for diverse lighting applications.^15–18^

Additionally, NPs have shown great promise in nanomedicine and biomedical imaging. RENPs exhibit unique properties, such as up-conversion, anti-Stokes shifts, high quantum efficiency, long luminescence lifetime, narrow emission spectra, and excellent colour purity, which make them ideal candidates for a range of medical applications.^19,20^ They are also employed as luminescent markers for diagnostics, therapeutic imaging, and biological labeling.^21^ Additionally, their ability to reduce UV-induced photo damage to biological samples and their resistance to photo-bleaching and photo-blinking offer distinct advantages for long-term imaging and monitoring of biological processes.^22–24^

In light of the growing importance of RENPs, this paper presents an extensive review of the literature, critically examining the various types of NPs, their synthesis, and applications across various fields. Through this review, we aim to provide a deeper understanding of the current state of research, explore the potential of these materials in diverse applications, and identify promising areas for future advancements in the development of RENP materials.

## 2. Synthesis of nanophosphors

In this section, we have explored various facets of NP synthesis, all of which are essential for optimizing the performance of the phosphor material.

### 2.1 Selection of phosphor host

**Fig. 3:**
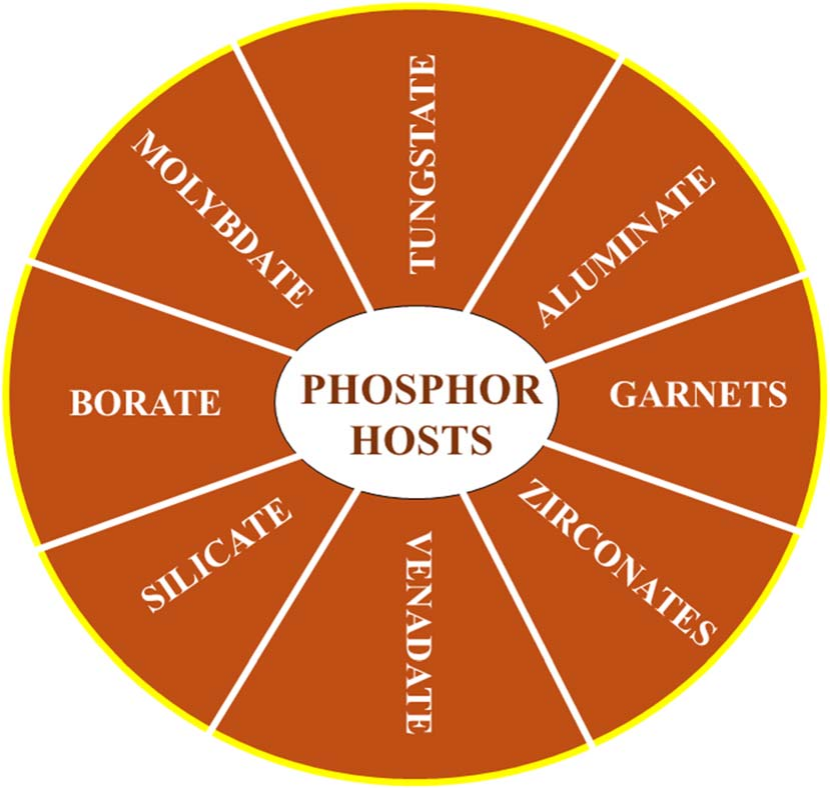
List of phosphor hosts.

A critical factor in the fabrication of phosphors is the selection of an appropriate host material. An ideal host should create an environment conducive to the efficient incorporation of dopant ions, thereby enhancing the material's performance for its intended application. Depending on the specific requirements of the application, the host material must exhibit excellent thermal, optical, and structural stability. Below, we present a summary of some of the most widely used phosphor hosts. A list of some of the prominent phosphor hosts is mentioned in Fig. 3.

#### 2.1.1 Tungstates

Tungstate compounds contain an oxoanion of tungsten or a mixed oxide incorporating tungsten. The simplest form of the tungstate ion is (WO_4_)^2−^, with ortho-tungstates featuring tetrahedrally coordinated W(vi) centers. These centers exhibit short W–O bond distances, typically around 1.79 angstroms. Tungsten itself is a greyish-white, lustrous metal, with an exceptionally high melting point of 3683 K, the lowest vapour pressure of any metal, and exhibits its peak tensile strength at 1650 °C. Furthermore, they also show low phonon energy and superior chemical and thermal stability. Due to these attributes, tungstate compounds are considered promising host materials for luminescence studies.^25,26^

#### 2.1.2 Vanadates

Vanadate serves as a robust host material, characterized by its oxoanion of vanadium in the maximum oxidation state of +5. The fundamental vanadate ion adopts a tetrahedral geometry. While its structure is often depicted with a double bond, this representation reflects only one resonance form. In reality, the vanadate ion exhibits a symmetrical tetrahedral configuration, wherein all four oxygen atoms are equivalently bonded to the central vanadium atom. Therefore, among the diverse range of host materials, alkaline earth metal vanadates activated by rare-earth elements emerge as particularly notable for their exceptional luminescent performance, spanning the visible to near-infrared (NIR) spectral regions. Furthermore, substituting vanadium with larger cations has been demonstrated to enhance both the luminescence efficiency and the thermal stability of these phosphors, making them highly suitable for advanced applications.^27–29^

#### 2.1.3 Aluminates

Aluminates have recently garnered significant attention due to their cost-effective and readily available raw materials. Notably, these materials can be tailored to exhibit desirable properties with ease. Among them, perovskite-type oxides doped with Mn^4+^ ions, such as LaAlO_3_ and GdAlO_3_, have been extensively studied as red phosphors due to their excellent chemical stability and high QE.^30,31^ LaAlO_3_, in particular, has attracted considerable interest not only as a red phosphor host but also for its applications in fields such as magnetic fluid generators, high-frequency capacitors, and superconducting substrates. For commercial applications, achieving uniform morphology, optimal particle size, and a narrow size distribution is crucial to enhancing luminescence performance. Furthermore, the development of aluminates extends beyond red phosphors to include applications in NIR, infrared, and persistent luminescent materials emitting in blue, green, and yellow regions. Their utility also spans afterglow and dosimetric phosphors, underscoring the ongoing advancements in optical materials and crystal engineering.^32–34^

#### 2.1.4 Borates

These minerals occur naturally and consist of boron, the fifth element in the periodic table, bonded with oxygen and other elements, thereby forming complex inorganic salts. Boron, with an ionic radius of 0.11 Å, can adopt both trigonal (BO_3_) and tetrahedral (BO_4_) coordination with oxygen.^35,36^ BO_3_ groups exhibit an average B–O bond valence of 1 valence unit, while BO_4_ groups average 3/4 valence unit, allowing them to polymerize *via* corner-sharing without violating the valence sum rule.^37^ This polymerization leads to diverse borate structures, including discrete polyanions forming clusters, chains, sheets, or frameworks.^38^ The dual coordination capability of boron enables a wide range of structural variations, resulting in hundreds of known borate crystal structures. However, only a few of these structural types hold significant practical relevance. Their ability to adopt three- or fourfold coordination, form a wide variety of structurally diverse compounds. Their intrinsic properties, such as a broad transparency range, large electronic bandgap, excellent thermal and chemical stability, low synthesis temperature, optical stability, strong nonlinear characteristics, and high optical damage threshold, make them ideal for optical materials.^39,40^ Their unique crystal structure enhances UV transparency, nonlinearity, and resistance to laser-induced damage.^41^ Current research focuses on synthesizing and characterizing borates for applications in lasers, nonlinear optics (NLO), and scintillators, highlighting their growing significance in advanced optical technologies.

#### 2.1.5 Silicates

Silicate matrices have gained significant attention for developing luminescent materials due to their excellent photoluminescence (PL) properties and chemical stability.^42^ Their composition, morphology, and size can be easily tailored to enhance PL performance, making them highly versatile for various applications.^43^ Silicate-based luminescent materials are valued for their low cost, high color rendering index (CRI), moisture resistance, and stability.^44^ In white light-emitting diodes (WLEDs), efficient and stable red-emitting silicates with low correlated colour temperature and high CRI are essential.^45,46^ Rare-earth disilicates and trisilicates phosphors are particularly noteworthy for their diverse applications across various domains, including nuclear medicine diagnostics, high-energy physics, positron emission tomography (PET), plasma and flat-panel display technologies, ionizing radiation detection, solid-state lighting, as well as gamma-ray and neutron excitation.^47,48^

#### 2.1.6 Phosphate

Phosphates encompass a class of compounds characterized by the presence of phosphorus-based oxyanions, ranging from the simple orthophosphate group to more complex chain, ring, and network structures.^49^ Additionally, oxyanions of phosphorus in reduced oxidation states, such as phosphite (HPO_3_^2−^), are also well-documented.^50^ The extensive diversity of phosphates arises from the variability in phosphate species, their ability to coordinate with a wide array of cations, and their interaction with other anions or molecules, particularly water.^51^ The chemical behaviour of phosphates exhibits notable parallels with that of solid silicates and borates. While much of the focus is directed toward phosphates containing metallic elements and smaller cations (*e.g.*, NH_4_^+^, H^+^), numerous phosphate salts also incorporate larger organic or inorganic coordination complex cations.^52,53^ Structurally, phosphates are typically rigid, chemically resilient, and, in their anhydrous forms, exhibit low solubility and high thermal stability.^54^ These features make them suitable for specialized applications such as immobilizing nuclear waste or serving as negative thermal expansion materials.^55^ Furthermore, many solid phosphate frameworks allow for the diffusion of extra-framework species, enabling their use as ion exchangers, ionic conductors, or microporous catalysts.^56^ Phosphate anions generally exhibit minimal absorption in the UV-vis spectrum, a property that positions solid phosphates as viable candidates for optical applications.^57^ This includes their use in the production of glasses, phosphors, nonlinear optical materials, and lasers, underscoring their multifaceted utility in advanced technological fields.^58,59^

### 2.2 Rare earth elements as the dopant

Rare earth (RE) elements constitute a group of 17 chemical elements in the periodic table, comprising the 15 lanthanides along with yttrium and scandium, which are included due to their analogous chemical behaviour.^60^ These elements predominantly exist in either the +2 or +3 oxidation states. RE ions exhibiting 4f^*N*^ → 4f^*N*^ transitions are characterized by superior coherence properties, highly resolved absorption lines, and long lifetimes. On the other hand, ions undergoing 4f^*N*^ → 4f^*N*−1^5d transitions demonstrate shorter lifetimes, broad absorption spectra spanning the ultraviolet to visible regions, and enhanced oscillator strengths.^61^ From the RE ions' spectroscopic properties, most RE elements are filled with one or more electrons in their outermost 4f orbital. Thus, different RE (trivalent/divalent ions) elements have abundant energy levels due to differences in their arrangement of 4f electrons. The spectroscopic characteristics of RE ions reveal that the majority of these elements possess one or more electrons occupying their outermost 4f orbitals. Consequently, both trivalent and divalent RE ions exhibit abundant energy levels, arising from the distinct configurations of their 4f electron arrangements.^62,63^Table 1 depicts the list of RE elements along with basic information, including atomic number, electronic configuration, and various oxidation states.

**Table 1:** List of RE elements along with electronic configuration and oxidation states.^64,65^

Rare earth element	Atomic number	Symbol	Electronic configuration	Oxidation states
Scandium	21	Sc	[Ar]3d^1^4s^2^	+3
Yttrium	39	Y	[Kr]4d^1^5s^2^	+3
Lanthanum	57	La	[Xe]4f^0^5d^1^6s^2^	+3
Cerium	58	Ce	[Xe]4f^1^5d^1^6s^2^	+3, +4
Praseodymium	59	Pm	[Xe]4f^3^6s^2^	+3, +4
Neodymium	60	Nd	[Xe]4f^4^6s^2^	+3, +4
Promethium	61	Pr	[Xe]4f^5^6s^2^	+3
Samarium	62	Sm	[Xe]4f^6^6s^2^	+2, +3
Europium	63	Eu	[Xe]4f^7^6s^2^	+2, +3
Gadolinium	64	Gd	[Xe]4f^7^5d^1^6s^2^	+3
Terbium	65	Tb	[Xe]4f^9^6s^2^	+3, +4
Dysprosium	66	Dy	[Xe]4f^10^6s^2^	+3, +4
Holmium	67	Ho	[Xe]4f^11^6s^2^	+3
Erbium	68	Er	[Xe]4f^12^6s^2^	+3
Thulium	69	Tm	[Xe]4f^13^6s^2^	+2, +3
Ytterbium	70	Yb	[Xe]4f^14^6s^2^	+2, +3
Lutetium	71	Lu	[Xe]4f^14^5d^1^6s^2^	+3

Furthermore, it was noted that the emission spectra of RE-doped phosphors exhibit minimal sensitivity to their chemical environment.^66^ This unique feature enables the fine-tuning of emission colors by introducing suitable dopants into the host lattice without necessitating alterations to the host material itself. The remarkably sharp line spectra characteristic of RE-doped phosphors arise from the low-lying, partially filled 4f subshell. These 4f electrons are effectively shielded by the outer 5p and 5s orbitals, resulting in a well-defined set of distinct energy levels.^67,68^

**Fig. 4:**
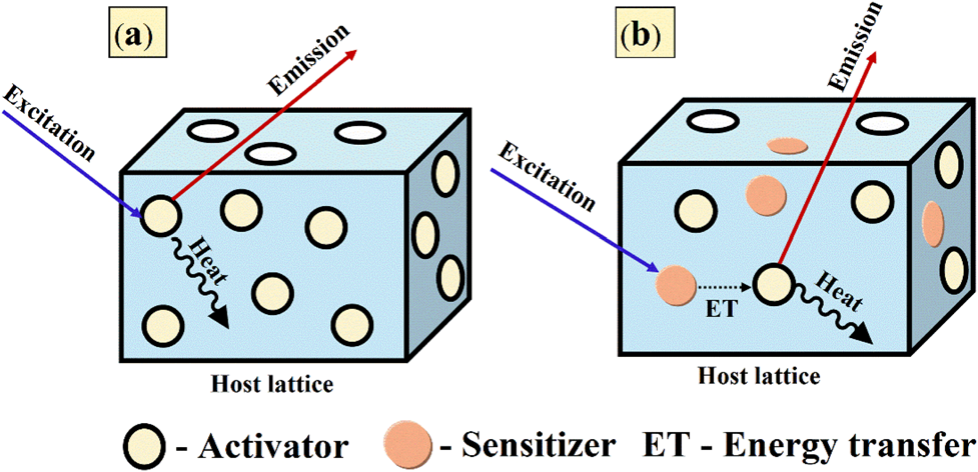
Luminescence mechanism in phosphors with (a) activator alone and (b) activator along with sensitizer.

When RE ions are introduced into a host lattice, the perturbations arising from the host induce the mixing of the host's even-parity p or d orbitals with the odd-parity f orbitals of the dopant ions. This interaction leads to a relaxation of the selection rules governing electronic transitions, thereby enhancing both the absorption efficiency and the emission yield of the doped system.^69,70^ Furthermore, the extent of relaxation is also contingent upon the symmetry of the environment surrounding the RE ions, with higher symmetry facilitating a greater relaxation effect.^71^ The excitation of RE-doped phosphors can occur either directly through the activator ion or indirectly *via* a sensitizer (A), which may itself be an RE ion, or through the incorporation of a dopant, termed a sensitizer (S). The sensitizer absorbs incident energy and subsequently transfers it to the RE activator, facilitating luminescence.^72,73^Fig. 4(a) and (b) represents the schematic representation of the luminescence mechanism within a phosphor host, highlighting the roles of both activator and sensitizer in the energy transfer process.

### 2.3 Synthesis methods

The investigation of advanced synthesis techniques continues to be a highly active research domain, facilitating the development of more efficient organic and inorganic phosphors. Below, we outline some of the most prominent methods employed for NP synthesis.

#### 2.3.1 Solid state reaction method

This method is one of the most widely used approaches for NP synthesis due to its ability to produce highly pure and crystalline products. It is also simple and cost-effective, making it suitable for industrial applications.^74,75^ In this process, precursor materials, typically in the form of oxides or carbonates, are combined in a stoichiometric ratio and subjected to high temperatures exceeding their decomposition point but remaining below the vaporization temperature, allowing the reaction to proceed in the solid state. At elevated temperatures, various chemical transformations occur, including solid-state ion exchange driven by atomic diffusion, as well as reduction and oxidation reactions, ultimately yielding the desired phosphor. After heating, controlled cooling is done to prevent phase segregation. However, this method has certain limitations, such as the requirement for high temperatures and limited control over morphology.^75^

#### 2.3.2 Sol–gel method

To mitigate the high-temperature requirements of phosphor synthesis, wet chemical approaches such as the sol–gel method offer a viable alternative. This method involves the uniform mixing of RE ions in nitrate, acetate, oxide, chloride, or alkoxide form with host material precursors, such as oxides or fluorides, in an aqueous or alcoholic medium.^76^ Hydrolysis agents, surfactants, and co-surfactants may be introduced to regulate and facilitate the reaction. The metal precursors are dissolved in alcohol or deionized water at 60 °C to 80 °C, with precise pH control to prevent unwanted precipitation and ensure the formation of a homogeneous gel, as pH significantly influences the phosphor properties. Hydrolysis initiates precursor breakdown, followed by condensation, leading to the formation of a gel network. As these reactions progress, polymeric chains develop, increasing the solution's viscosity. The resulting gel is dried at 150 °C to 200 °C to remove residual solvents and impurities, then annealed at 400 °C to 800 °C to obtain a well-crystallized, pure-phase material. Optimizing synthesis time is crucial, as prolonged reactions can lead to larger aggregate formation, potentially affecting the phosphor's performance.^77^

#### 2.3.3 Solution combustion method

This is an auto-ignition technique for the NP synthesis method, relying on a highly exothermic reaction between a fuel (*e.g.*, sucrose, glycine, urea) and an oxidizer, typically RE metal nitrates. The precursors are combined in a stoichiometric ratio and ignited, triggering a self-sustaining combustion process. This reaction releases gases such as NO_2_, CO_2_, and N_2_, resulting in the formation of a foamy solid. Notably, once initiated, the reaction proceeds independently without the need for continuous external energy input. During combustion, the high temperature and rapid reaction kinetics facilitate the uniform size distribution.^78,79^ However, controlling the exothermic reaction remains a significant challenge, as uncontrolled combustion can lead to reduced crystallinity. Additionally, the method is constrained to specific material compositions, and the use of combustible reactants necessitates stringent safety precautions. Despite these limitations, solution combustion synthesis remains a straightforward and efficient approach for producing NPs with well-defined properties.^80^

#### 2.3.4 Hydrothermal method

This method is a variant of wet chemical synthesis, where precursor solutions are heated in an autoclave beyond the solvent's boiling point. The elevated temperature, typically around 300 °C, generates high internal pressure, leading to the formation of a supersaturated solution. This highly concentrated environment promotes hydrolysis, condensation, and crystallization, facilitating NP formation. Once the reaction is complete, the autoclave is gradually cooled to room temperature, allowing the phosphor particles to precipitate.^81^ This synthesis technique offers several advantages, including precise control over crystal growth and high-purity product formation. The elevated pressure conditions enable better regulation of both morphology and crystallinity, leading to well-defined nanoparticle structures. However, compared to other synthesis methods, this approach is more complex, time-intensive, and necessitates specialized equipment. These factors limit its feasibility for large-scale production, making it less suitable for industrial manufacturing.^82,83^

#### 2.3.5 Co-precipitation method

This method is widely utilized for phosphor synthesis, particularly in large-scale industrial applications, due to its scalability. Additionally, it is a cost-effective approach that enables the production of phosphors with uniform morphology and a well-distributed particle size.^84^ In this process, stoichiometric amounts of inorganic salts of the host material and RE dopants are dissolved in a suitable solvent and thoroughly mixed using a magnetic stirrer at approximately 60 °C. A precipitating agent is then gradually introduced dropwise into the solution, facilitating the formation of an insoluble compound. The resulting mixture is allowed to stand undisturbed for approximately 10–20 hours to ensure complete precipitation, yielding the desired phosphor material. To enhance the purity of the phosphor, additional washing and calcination may be necessary. Moreover, inadequate precipitation and insufficient washing can result in non-uniform particle formation, emphasizing the importance of process optimization to achieve consistent and high-quality phosphor materials.^85,86^

## 3. Applications of nanophosphors

### 3.1 Lighting applications

**Fig. 5:**
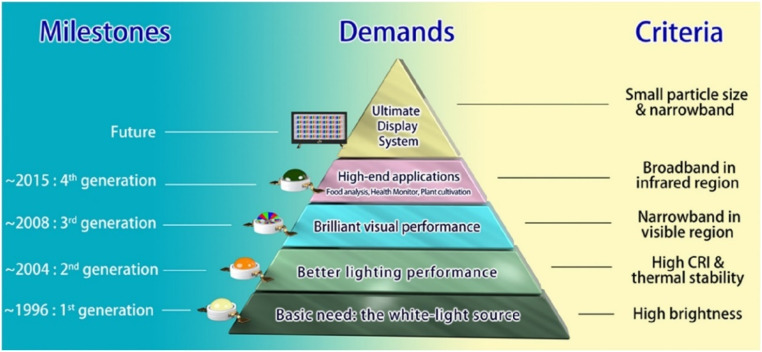
Milestones in phosphor development reflecting demands from basic white-light lighting to advanced applications. Reprinted with permission from ref. 87 copyright 2022, American Chemical Society.

Solid-state lighting (SSL) technology, encompassing LEDs and organic LEDs (OLEDs), has revolutionized the lighting industry. Its advancement is driven by the increasing demand for energy-efficient solutions and severe environmental requirements. SSL has widely utilized phosphors doped with RE ions. Fig. 5 presents the milestones in phosphor material development along with the defining criteria for each generation.^87^ However, there is still significant scope for further research in this field, with a primary focus on reducing electrical energy consumption.

To achieve efficient white light emission and cover the visible spectrum, the following key factors must be considered to enhance performance and effectiveness.

(a) Using a single host material with multiple RE activators reduces WLED costs and simplifies synthesis.

(b) The colour rendering index (CRI) evaluates a light source's ability to accurately reproduce the colours of illuminated objects. CRI values range from 55 to 65, considered to be fair, and 65–75 are categorized as good, and values above 75 represent an excellent light source.

(c) Correlated color temperature (CCT) is a key parameter that defines the colour appearance of a light source and is measured in kelvin. Lower CCT values, around 2000 K to 3000 K, produce a warm light used in residential lighting, while higher values, ranging from 4000 K to 6500 K, create a cooler, daylight-like illumination suitable for commercial and industrial applications.

(d) Phosphor materials should exhibit thermal stability to withstand temperature variations. Additionally, they must possess chemical stability to enhance synthesis efficiency and ensure the formation of desired compounds.

(e) Finally, the phosphor should exhibit a high quantum yield (QY), defined as the ratio of emitted photons to absorbed photons, ensuring efficient light conversion.

Some of the notable works in the field of lighting applications using NPs are discussed in this section. In certain instances, the host matrix was held constant while exploring the luminescent behaviour under varying conditions, specifically, through single doping, co-doping, and the incorporation of charge compensators. Conversely, in other studies, the dopant ions were kept fixed while different host lattices were investigated to enable a comparative analysis of their influence on key optical and structural properties.

To start with, Saha *et al.* studied the luminescence behaviour of Li^+^ and Eu^3+^ co-doped nano magnesium aluminate (MgAl_2_O_4_) phosphors prepared *via* the sol–gel method.^88^ When doped solely with Eu^3+^, the substitution of Mg^2+^ by Eu^3+^ led to a net positive charge due to their differing valence states, creating vacancies that act as luminescence quenchers.^89^ To address this issue, they used Li^+^ as a charge compensator and co-doped it into optimized MgAl_2_O_4_: Eu^3+^. The ideal Eu^3+^ concentration was 2 mol%, while Li^+^ content varied from 0.5 to 2.5 mol%, keeping Eu^3+^ constant. Spectroscopic analysis revealed that the incorporation of Li^+^ ions resulted in a twofold enhancement of PL intensity, with the peak intensity achieved at a Li^+^ concentration of 2 mol%. Moreover, the integration of Li^+^ was found to enhance the QE by increasing the probability of radiative transitions within the system. The study does not examine thermal quenching behaviour, which is vital for evaluating phosphor performance under high-temperature LED operation. Also, the stability under prolonged UV exposure was not tested, which is critical for real-world lighting and display systems. Whereas Manjula *et al.* used a low-temperature solution combustion method with oxalyl dihydrazide (ODH) as fuel to prepare MgAl_2_O_4_:Eu^3+^ nanophosphors.^90^ They found that the optimum Eu^3+^ concentration was 5 mol% and concluded that the dipole–dipole interaction was the dominant mechanism for concentration quenching. The obtained band gap of the pure sample was 5.1 eV using DFT calculations, whereas using DRS measurements, it was found to vary between 4.6 eV to 4.9 eV for the doped samples.

**Fig. 6:**
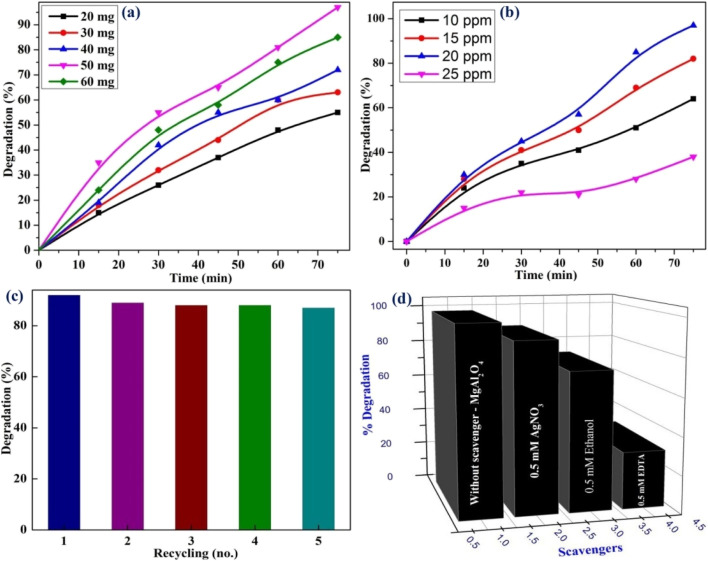
(a) Photodegradation rate of the dye (b) effect of dye concentration on degradation rate (c) recyclability and stability of MgAl_2_O_4_ (d) scavenging examinations. The figures were reproduced from ref. 93 with copyright permission from John Wiley and Sons.

Additionally, they carried out photocatalytic measurements, which were missing from the works of Saha *et al.* They demonstrated effective photocatalytic performance in degrading FOR dye under UV light, achieving an absorbance of 96% after 135 minutes of illumination. The MgAl_2_O_4_ photocatalyst maintained consistent activity over five consecutive cycles, indicating good recycling stability. To determine the active species involved in the photocatalytic decolouration, they used AgNO_3_, alcohol, and ethylenediaminetetraacetic acid (EDTA) as the different scavengers. The outcomes of the rate of photodegradation of dye along with various concentrations, recycling stability, and scavenging examinations are presented in Fig. 6(a)–(d). Therefore, it can be concluded that the significant reduction in efficiency upon EDTA addition confirms that holes (h^+^) play the dominant role in the photocatalytic decolouration mechanism. Recently, Halefoglu *et al.* and Lamonova *et al.* have explored the luminescent characteristics of MgAl_2_O_4_ phosphors doped with Sm^3+^ and Tb^3+^ ions, respectively. However, their investigations did not consider the influence of charge compensating agents, nor did they provide a comprehensive analysis of the thermal behaviour of the synthesized materials, leaving important aspects of phosphor performance underexplored.^91,92^ Next, Panigrahi *et al.* reported the co-doped MgAl_2_O_4_:Eu^3+^,Dy^3+^ nanophosphors, prepared *via* a modified Pechini-type sol–gel method.^93^ Owing to the proximity in energy levels between the ^4^F_9/2_ state of Dy^3+^ and the ^5^D_0_ state of Eu^3+^, resonant energy transfer (ET) occurs through a multipolar interaction mechanism.^94^ To explore tunable emission properties, two series of samples were synthesized: Mg_(1−*x*−0.02)_Al_2_O_4_:0.02Dy^3+^,*x*Eu^3+^ (*x* = 0.1, 0.2, 0.3, 0.4, and 0.5 mol%) and Mg_(1−*y*−0.02)_Al_2_O_4_:0.02Eu^3+^,*y*Dy^3+^ (*y* = 0.2, 0.3, 0.4, 0.5, and 1 mol%). The PL emission spectra of the samples are recorded under 351 nm excitation, revealing an intriguing trend: while maintaining Dy^3+^ concentration at 2 mol%, the intensities of Dy^3+^ emissions at 488 nm and 575 nm diminished progressively with an increase in Eu^3+^ concentration (0.1–0.5%), accompanied by a simultaneous intensification of Eu^3+^ emission peaks. Conversely, fixing Eu^3+^ concentration while incrementally increasing Dy^3+^ concentration unexpectedly led to a substantial enhancement of Eu^3+^ emission intensity. Among the synthesized phosphors, NPs co-doped with 2 mol% Dy^3+^ and 0.2 mol% Eu^3+^ exhibited superior white light emission under UV excitation, with chromaticity coordinates of (0.31, 0.33) aligning closely with white light.^95^ Additionally, the NPs demonstrated a remarkably high absolute quantum yield of nearly 67% and a CCT value of 6494 K. These findings establish MgAl_2_O_4_:2% Dy^3+^,0.2% Eu^3+^ as a highly promising candidate for WLED applications. Despite extensive studies on RE-doped MgAl_2_O_4_ nanophosphors, a significant gap remains in understanding their PL behavior at elevated temperatures. In particular, the thermal quenching characteristics have not been thoroughly explored. To substantiate the potential of these materials for high-performance LED applications, it is imperative to conduct temperature-dependent PL analyses to accurately determine the quenching temperature (*T*_Q_). Such insights are essential for assessing their thermal stability and operational reliability under real-world device conditions.

Shivram *et al.* reported the synthesis of CaTiO_3_:Eu^3+^ nanophosphors using a low-temperature solution combustion method, achieving a crystallite size of approximately 40–45 nm.^96^ Under 398 nm excitation, a strong red emission centered at 615 nm was observed, attributed to the hypersensitive ^5^D_0_ → ^7^F_2_ transition of Eu^3+^. The optimal dopant concentration was identified as 3 mol%, and the corresponding CIE chromaticity coordinates closely matched the NTSC red standards, making the phosphor suitable for LED applications. In a separate study, Mazzo *et al.* synthesized CaTiO_3_:Eu^3+^ nanophosphors *via* a polymeric precursor route, yielding smaller particle sizes in the range of 24–35 nm.^97^ They determined the optimal Eu^3+^ concentration to be 1.5 mol%, beyond which concentration quenching set in. Furthermore, annealing was found to enhance the optical band gap due to lattice reorganization at elevated temperatures. Despite these promising findings, both studies lacked evaluation of temperature-dependent PL and thermal quenching, which are critical for real-world LED reliability. Additionally, neither study included an analysis of colour purity, decay dynamics, or lifetime measurements. Some of these limitations were addressed by Singh *et al.*, who employed a chemical co-precipitation method to prepare CaTiO_3_:Eu^3+^ nanophosphors.^98^ Their study identified an optimal Eu^3+^ concentration of 5 mol%, with quenching attributed to dipole–quadrupole interactions. The optimized phosphor exhibited a quantum efficiency of 17.46%, correlated color temperatures (CCT) between 1740 and 2522 K, and CIE coordinates of (0.63, 0.37), confirming its potential as a red-emitting phosphor for domestic lighting. Further advancements were made by Sasidharan *et al.*, who investigated Y^3+^ co-doping in CaTiO_3_:Eu^3+^ systems. By fixing the Eu^3+^ concentration at 7 mol%, they found that co-doping with 5 mol% Y^3+^ enhanced the red emission intensity at 614 nm by a factor of 10 compared to Eu-only samples.^99^ This enhancement was accompanied by a notable increase in colour purity (from 82.9% to 91.4%), a maximum luminescence lifetime of 1.314 ms, and a peak internal quantum efficiency of 81.6%, as confirmed *via* Judd–Ofelt analysis. In 2017, Singh and Manam synthesized CaTiO_3_:Dy^3+^ nanophosphors using a solid-state reaction method and demonstrated efficient blue and yellow emissions under 386 nm UV excitation, with optimal luminescence at 4 mol% of dopant.^100^ This composition yielded near-white CIE coordinates (0.28, 0.32) and a highly correlated color temperature of 9222 K, indicating promise for cold white light applications. The study also highlighted the role of dipole–dipole interactions in concentration quenching and reported moderate thermal stability. Building on this, the 2021 work by Shanbhag *et al.* introduced Li^+^ co-doping and SiO_2_ core–shell coating *via* solution combustion synthesis, achieving significantly enhanced luminescence and quantum efficiency (∼93%) with retained emission characteristics.^101^ The incorporation of Li^+^ (0.5 mol%) and the SiO_2_ shell effectively reduced non-radiative surface defects and improved crystallinity, leading to higher color purity (∼92%) and optimal CCT (6532 K). Despite the advancements, critical aspects of the material system remain insufficiently addressed. Specifically, the role and mechanism of Li^+^ ions in facilitating charge compensation and lattice incorporation lack comprehensive elucidation through advanced spectroscopic techniques such as X-ray photoelectron spectroscopy (XPS). Additionally, the atomic-level interactions between the SiO_2_ shell and the CaTiO_3_ host matrix have not been thoroughly investigated, limiting our understanding of the interface chemistry and its influence on luminescence efficiency. Moreover, long-term stability assessments, including photostability, thermal endurance under continuous excitation, and integration into functional LED prototypes, are absent from the current literature. While the contributions of Singh *et al.* and Sasidharan *et al.* have addressed foundational aspects such as structural characterization and luminescence optimization, essential parameters, including the effects of charge compensators, material reusability, and operational reliability under high-power excitation, remain unexplored. These factors are critical for the practical deployment of CaTiO_3_-based nanophosphors in robust, high-performance solid-state lighting applications.

Next, Kumar *et al.* explored the structural and photoluminescent characteristics of RE-doped GdSr_2_AlO_5_ nanophosphors, synthesized *via* a low-cost, urea-assisted gel combustion method.^102–104^ The materials doped individually with Dy^3+^, Eu^3+^, and Sm^3+^ ions are tailored for WLED applications, aiming to overcome the limitations of conventional lighting materials such as poor colour rendering and the absence of full-spectrum emission. All synthesized phosphors retained a well-defined tetragonal crystal structure, verified through XRD and Rietveld refinement. SEM and transmission electron TEM confirmed homogeneous, nanometer-sized particles (typically 31–35 nm). Each dopant imparted distinct emission properties: Dy^3+^ (optimal at 3 mol%) exhibited characteristic blue (488 nm), yellow (582 nm), and red (669 nm) emission bands arising from intra-4f transitions, producing a balanced white light when excited at 351 or 272 nm. Eu^3+^ (also optimal at 3 mol%) showed dominant red emission at 612 nm due to the hypersensitive ^5^D_0_ → ^7^F_2_ transition under 268 nm excitation, making it suitable for generating warm white light. Sm^3+^ (optimal at 4 mol%) emitted strong orange-red light centered at 603 nm under 273 nm excitation, primarily from the ^4^G_5/2_ → ^6^H_7/2_ transition. All systems demonstrated effective energy transfer from the Gd^3+^ host to the respective RE ions, a key factor in enhancing emission intensity. Notably, concentration quenching beyond the optimal dopant level was observed, attributed to non-radiative energy losses *via* dipole–dipole or quadrupole–quadrupole interactions among neighbouring activator ions. Judd–Ofelt analysis and lifetime measurements were conducted in detail for the Eu^3+^ system, yielding a quantum efficiency of 67.62% and confirming a favourable asymmetric environment around Eu^3+^ ions. Thermal stability for Eu^3+^ was also assessed, with a thermal quenching activation energy of 0.216 eV, suggesting strong potential for practical LED integration. In contrast, the Dy^3+^ and Sm^3+^ doped systems lacked equivalent thermal and quantum efficiency analyses, presenting an opportunity for further study. Despite the promising outcomes, thermal quenching behaviour and long-term photostability were only partially addressed, especially lacking in Dy^3+^ and Sm^3+^ doped systems. Critical parameters for commercial viability, such as Colour Rendering Index (CRI), CCT, and luminous efficacy, were not quantitatively evaluated. Additionally, while the excitation was primarily conducted in the UV region, behavior under standard blue LED excitation (around 450 nm), which is common in current WLED technology, remains unexplored. There is also untapped potential in exploring various RE doping and co-doping strategies (*e.g.*, Dy^3+^/Sm^3+^ or Eu^3+^/Sm^3+^) that could enable more flexible colour tuning and mitigate concentration quenching. Hooda *et al.* focused on BaYZn_3_AlO_7_ phosphors doped with Eu^3+^ and Er^3+^ ions, employing solution combustion synthesis to achieve red and yellowish-green emissions, respectively.^105,106^ In a parallel study, Kaushik *et al.* synthesized Tb^3+^ doped BaYZn_3_AlO_7_ nanophosphors using the same combustion-based technique, targeting efficient green emission.^107^ Each dopant induces distinct emission colours. Eu^3+^ yields strong red emission at 611 nm, Er^3+^ produces yellowish-green emission at 527 and 566 nm, and Tb^3+^ exhibits green emission centered at 543 nm, highlighting the tunability of the host lattice for solid-state lighting applications, especially in warm and white LEDs. Morphologically, the nanoparticles ranged from 36 nm to 86 nm and showed characteristic porous, agglomerated features typical of combustion-synthesized materials. While the Eu^3+^ doped sample was thoroughly analyzed using Judd–Ofelt theory to explain its luminescence mechanism and quenching behavior, such detailed energy transfer studies were absent in the Er^3+^ and Tb^3+^ studies. Additionally, quantum efficiency was reported only for the Tb^3+^ system (78%), with no corresponding data for the Eu^3+^ and Er^3+^ systems. A significant gap across all studies is the lack of long-term photostability data and comparative synthesis methods to evaluate performance in practical scenarios. Future work should explore co-doping strategies, thermal degradation studies, and integration into functional devices to fully realize the potential of these nanophosphors in commercial lighting technologies. Next, the sequence of research by Phogat *et al.* (2021), Devi *et al.* (2023), and Solanki *et al.* (2025) presented a coherent and progressive development of Eu^3+^ doped vanadate nanophosphors, focusing on enhancing red emission for solid-state lighting applications.^108–110^ In the foundational work by Phogat *et al.* Ca_9_Bi(VO_4_)_7_:Eu^3+^ nanophosphors were synthesized *via* a solution combustion method, demonstrating an intense red emission at 614 nm, and a high quantum efficiency at 70 mol% Eu^3+^, making it a strong candidate for near-UV WLEDs.^108^ This research emphasized structural refinement using Rietveld analysis and photoluminescent behaviour characterized through Judd–Ofelt theory. Building on these findings, Devi *et al.* replaced Bi^3+^ with Y^3+^ in the host matrix, resulting in Ca_9_Y(VO_4_)_7_:Eu^3+^ nanophosphors with an even higher quantum efficiency (65.7%) and maintained strong emission at 619 nm.^109^ Their study also incorporated Dexter's theory and the Inokuti–Hirayama model to detail dipole–dipole interactions responsible for concentration quenching, thus offering deeper insight into energy transfer mechanisms. Extending this trajectory, Solanki *et al.* replaced Y^3+^ with La^3+^, synthesizing Ca_9_La(VO_4_)_7_:Eu^3+^ nanophosphors that not only sustained high photoluminescent performance (emission at 615 nm, QE ∼ 58.67%) but also pioneered their application in latent fingerprint detection (LFP), showcasing dual-use functionality for both lighting and forensic technologies.^110^ Furthermore, Phogat *et al.* also extended their analysis to other dopants also.^111,112^ They prepared Ca_9_Bi(VO_4_)_7_:Sm^3+^ nanophosphors synthesized *via* solution combustion, and they observed intense orange-red emission centered around 600–610 nm due to the ^4^G_5/2_ → ^6^H_7/2_ transition.^111^ This composition achieved high quantum efficiency (∼81%) and revealed efficient energy transfer from the VO_4_^3−^ group to Sm^3+^ ions. Dalal *et al.* substituted Bi^3+^ with Gd^3+^, fabricating Ca_9_Gd(VO_4_)_7_:Sm^3+^ phosphors.^113^ This study achieved improved thermal stability, quantum efficiency (82.84%), and extended emission in the reddish-orange region (606 nm), facilitated by the substitution of slightly smaller Gd^3+^ ions with Sm^3+^, resulting in minimal structural distortion and a narrow bandgap (3.66 eV), enhancing radiative transitions. Furthermore, they also explored the Ca_9_La(VO_4_)_7_:Sm^3+^ system, reporting intense orange-red luminescence with 10 mol% Sm^3+^ as the optimal doping level.^114^ The substitution of La^3+^ with ionic properties similar to Sm^3+^ was structurally favourable and resulted in a robust trigonal framework. The optimized sample displayed a slightly reduced bandgap (3.42 eV), increased emission intensity, and favourable chromaticity coordinates (0.5868, 0.4027), confirming its application potential in modern lighting technologies. Across all three works, the solution combustion method remained a consistent, low-cost, and energy-efficient synthesis route. Additionally, an extensive overview of optical studies of various RENPs has been systematically compiled in Table 2.

**Table 2:** List of recently reported nanophosphors and their optical properties^a^

Nanophosphor	Synthesis method	*λ*_ex_ (nm)	*λ*_emax_ (nm)	CCT (K)	CP (%)	Ref.
Y_4_Al_2_O_9_:Sm^3+^	Gel combustion	406	602	1664–1764	87.4–92.1	115
Y_4_Al_2_O_9_:Dy^3+^	Combustion	348	483	5037.88–6609.22	—	116
SrGdAlO_4_:Sm^3+^	Solution combustion	407	600	—	—	117
Ba_3_Y_4_O_9_:Tb^3+^	Solution combustion	292	544	—	—	118
SrLaAlO_4_:Dy^3+^	Solution combustion	352	484	7.4 × 10^3^ to 11.1 × 10^3^	23.02–39.27	119
BaSrGd_4_O_8_:Dy^3+^	Solution combustion	352	570	1714–2700	—	120
GdAlO_3_:Er^3+^	Gel combustion	377	546	5749–6005	76.66–90.44	121
Ca_9_La(PO_4_)_7_:Dy^3+^	Solution combustion	352	485	6973–18 425	11.30–36.5	122
GdAl_2_O_9_:Er^3+^	Solution combustion	382	558	5983–7169	—	123
NaCaVO_4_:Dy^3+^	Combustion	310	579	3306–3369	50.80–52.30	124
KSrVO_4_:Sm^3+^	Combustion	402	600	1645	—	125

a *λ*_ex_ – excitation wavelength, *λ*_emax_ – maximum emission wavelength, CP – colour purity.

#### 3.1.1 Limitations and challenges

Despite the extensive development of phosphors with high QE, only a limited number have successfully transitioned into commercial use in phosphor-converted white light-emitting diodes (pc-WLEDs). Consequently, current research focuses not merely on achieving high QE but on developing materials that also meet essential application-specific criteria, including high CRI and CCT for both cool and warm white light emissions. Long-term operational stability over 50 000 hours is critical for competing with commercial standards such as YAG:Ce^3+^. The growing demand for high-power pc-WLEDs presents additional challenges, such as efficiency in LED chips, luminescence saturation of phosphors, and thermal degradation at elevated temperatures (∼200 °C). While YAG:Ce^3+^ remains effective as a yellow-emitting phosphor, alternatives like the red-emitting KSF:Mn^4+^ show performance limitations, particularly saturation at input intensities exceeding 0.1 W mm^−2^. Thus, identifying robust red phosphors for high-power applications remains a key research goal.^126^ Furthermore, the reliance on blue InGaN chips in current pc-WLEDs raises health concerns due to potential overexposure to blue light, prompting interest in ultraviolet (UV) or violet chip-based systems. These systems typically incorporate either a broadband white-light-emitting phosphor or a combination of red, green, and blue phosphors. However, limitations such as lower chip efficiency and large Stokes shifts reduce the overall efficacy of UV/violet-excited pc-WLEDs. Nevertheless, these systems are promising for applications requiring warm, low-intensity lighting, such as nighttime illumination. Integrating machine learning approaches is proving effective in predicting materials with optimal combinations of structural and optical properties.^127,128^

### 3.2 Sensing applications

**Fig. 7:**
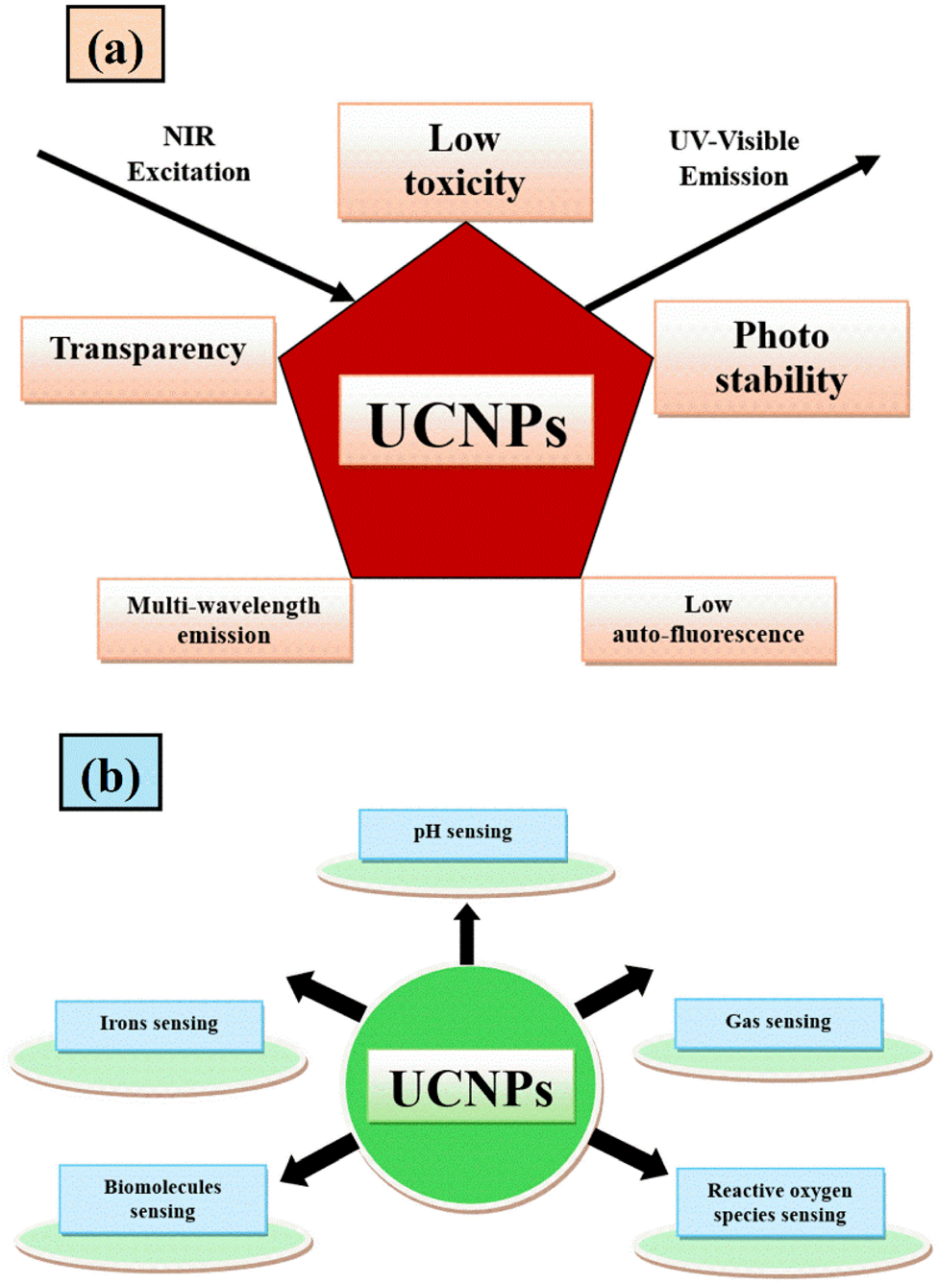
(a) Properties of UCNPs (b) various sensing applications of UCNPs.

RENPs have gathered significant attention for sensing applications due to their unique optical properties. These phosphors, doped with RE ions, exhibit tunable emission spectra under specific excitation conditions, enabling their use in temperature, pressure, and gas sensors. Furthermore, UCNPs provide enhanced sensitivity and signal reliability, particularly for remote and non-contact sensing applications. Additionally, the long luminescence lifetime and low energy loss of these phosphors make them ideal candidates for real-time monitoring and detection in various environments. Fig. 7(a) and (b) illustrate the distinctive optical properties of UCNPs and their corresponding sensing applications, respectively.^129^ Some of the most prominent advancements within this domain are presented below.

To assess the suitability of the prepared NPs for sensing applications, it is essential to consider several key parameters that influence their performance. The following section outlines the critical factors that determine the effectiveness of NPs in metal ion and temperature sensing applications.

#### 3.2.1 Metal ion sensing

To assess the sensing characteristics of the NPs toward various metal cations, aqueous solutions of different metal ions were prepared at a given concentration.^130^ Each metal ion solution was separately mixed with the nanomaterial in an aqueous medium at a pH equal to 7. By making use of the Stern–Volmer equation given below, the metal ion can be sensed^130^
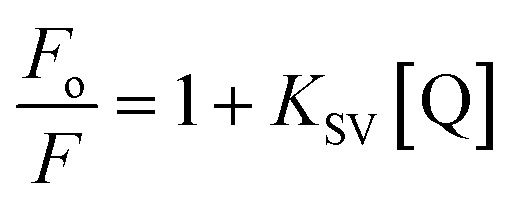
Here, *F*_o_ and *F* represent the fluorescence intensities of phosphor before and after the presence of metal ions, respectively. [Q] denotes the concentration of the metal ion, while *K*_SV_ is the Stern–Volmer constant. Some of the important parameters in the case of metal ion sensing include.

##### (a) The detection limit (LOD)

LOD refers to the lowest concentration of a metal ion that can be reliably detected, and it is given by^131^
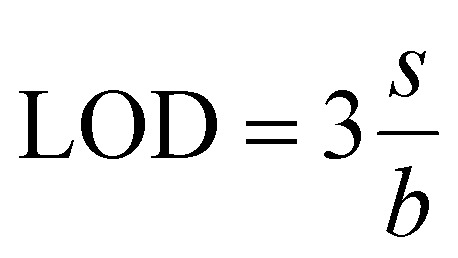
Here, *s* represents the standard deviation, while *b* denotes the slope of the calibration curve.

##### (b) Quantification detection limits (LOQ)

It is defined as the lowest concentration of a metal ion that can be accurately quantified with a reasonable degree of precision and accuracy. It is given by^132^
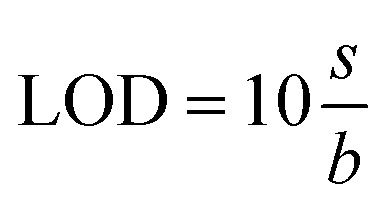


##### (c) Half-quenching concentration (*C*_0.5_)

Half-quenching concentration is defined as the concentration of metal ions at which the fluorescence intensity reduces to half of its initial value (*F*_o_/2). On substituting this condition in the Stern–Volmer equation and simplifying, we get
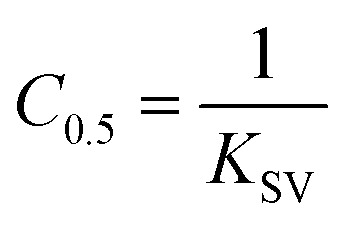


Below we have listed some of the important works in the field of phosphor-based metal ion sensors, including the detection of Cd^2+^, Pb^2+^, Fe^3+^, As^3+^, and Cr^3+^ ions using various phosphor systems. The two studies by Tashi *et al.* and Saif *et al.* present distinct approaches to the development of lanthanide-doped nanophosphors for heavy metal ion sensing. Tashi *et al.* hydrothermally synthesized NaGdF_4_ nanophosphor co-doped with Eu^3+^ and Ce^3+^ for chemical sensing of heavy metal ions (Cd^2+^, Pb^2+^, and Cr^3+^) in wastewater.^133^ These NPs exhibit an energy transfer from Ce^3+^ to Gd^3+^ to Eu^3+^, allowing them to act as a sensitive probe under UV excitation. Although the study includes structural and spectroscopic characterizations and indicates successful detection of Cr^3+^, Pb^2+^, and Cd^2+^, it lacks specific analytical performance metrics such as detection limits, response time, or interference from other ions. Furthermore, its application is limited to controlled laboratory settings, without validation using actual environmental samples. In contrast, the study by Saif *et al.* synthesized BaZrO_3_:Eu^3+^ nanophosphor for the detection of chromium ions from tannery leather and wastewater.^134^ They presented a more targeted and application-focused approach. The BaZrO_3_:Eu^3+^ nanophosphor, synthesized using a sonochemical sol–gel method, exhibits strong and stable red fluorescence that is selectively quenched in the presence of Cr^3+^ ions. Unlike the broader scope of Tashi *et al.*, Saif *et al.* focus solely on Cr^3+^ detection but provide a comprehensive evaluation, including LOD = 3.8 × 10^−9^ mol L^−1^, a high *K*_SV_ value of 7.05327 × 10^7^ mol L^−1^, and excellent performance in real tannery leather and wastewater samples. The study confirms that quenching occurs *via* a static coulombic interaction, substantiated by lifetime and absorption-emission overlap studies. Tashi *et al.* exploit a multi-ion sensitization mechanism for enhanced luminescence and multi-ion sensing, and Saif *et al.* demonstrate the practical utility of their sensor with rigorous analytical validation. The work by Tashi *et al.* lacks real-world testing, quantitative sensitivity data, and interference analysis. Therefore, the NaGdF_4_:Eu^3+^/Ce^3+^ system could be further investigated for its potential selectivity and comparative performance against the BaZrO_3_:Eu^3+^ system when sensing Cr^3+^. Next, we transition into a comparison of two distinct approaches to Fe^3+^ ion sensing using lanthanide-doped nanophosphors. In the first study, Mahmoud *et al.* developed a multifunctional Bi_12_SiO_20_:Pr^3+^ nanophosphor *via* a hydrothermal method.^135^ This material exhibits a strong pinkish-red emission under UVA light and demonstrates versatility across three key applications: latent fingerprint visualization, anti-counterfeiting ink formulation, and Fe^3+^ ion detection in drinking water. The study offers a thorough performance analysis, with a Stern–Volmer quenching response over the concentration range of 0 to 17 × 10^−5^ mol L^−1^ and a detection limit of 1.56 × 10^−5^ mol L^−1^. The nanophosphor also displays high selectivity toward Fe^3+^ ions, as evidenced by pronounced fluorescence quenching under UV exposure. Notably, real sample testing in drinking water revealed high recovery rates and excellent correlation with standard atomic absorption spectroscopy, further establishing the material's practical applicability. They also examined the PL lifetimes and morphology in detail, contributing valuable insight into the sensor's stability and efficiency under varying environmental conditions. A more recent study by Singhaal *et al.* introduced a polyethylenimine (PEI)-functionalized NaCeF_4_:Tb^3+^/Eu^3+^ nanophosphor, also synthesized hydrothermally.^136^ This system functions as a dual-mode sensor for Fe^3+^ ions and picric acid (PA). The PEI layer enhances water dispersibility and promotes complexation with analytes, enabling selective quenching of the Eu^3+^ emission, especially at the 594 nm peak. The material demonstrated a low detection limit of 1.39 ppm for Fe^3+^ and a high *K*_SV_ value of 3.8 × 10^4^ M^−1^, indicating both sensitivity and selectivity. A distinctive feature of this study is the integration of reduced graphene oxide (RGO), used to explore energy transfer processes and further fine-tune PL properties. Together, these studies exemplify the diversity in material design and sensing strategy within Fe^3+^ ion detection. Mahmoud *et al.* emphasize practical applicability and multifunctional use, while Singhaal *et al.* offer an advanced, tunable platform with potential for future expansion. Furthermore, Kumar and Roy presented a highly sensitive and selective luminescence-based method for detecting As^3+^ in water using Eu^3+^ doped GdVO_4_ nanophosphors synthesized *via* a hydrothermal method.^137^ These nanophosphors exhibit strong red emission due to energy transfer from VO_4_^3−^ groups to Eu^3+^ ions, and their fluorescence is quenched in the presence of As^3+^ due to covalent bonding with surface Eu–OH groups, causing nanoparticle aggregation. The system showed an excellent LOD of 39 nM, well below the WHO permissible limit of 130 nM, and was tested successfully in real tap and river water samples with high accuracy and minimal interference from other ions. This robust selectivity across varying pH levels and fast response time of 30 seconds underscores its potential for practical field use in environmental monitoring.

#### 3.2.2 Limitations and challenges

The collective findings from these studies demonstrate the growing potential of lanthanide-doped nanophosphors as powerful platforms for selective and sensitive detection of heavy metal ions in environmental matrices. Each study presents a unique material system and sensing strategy tailored to specific target ions (*e.g.* Cr^3+^, Fe^3+^and As^3+^), highlighting both the strengths and areas of opportunity in NP-based sensor development. They also reveal critical gaps such as the need for consistent real-world validation, interference studies, and multifunctional capabilities. Moving forward, a systematic approach that integrates high-performance material engineering with field-deployable validation and compatibility will be essential for advancing these systems toward commercial platforms.

#### 3.2.3 Temperature sensing

**Fig. 8:**
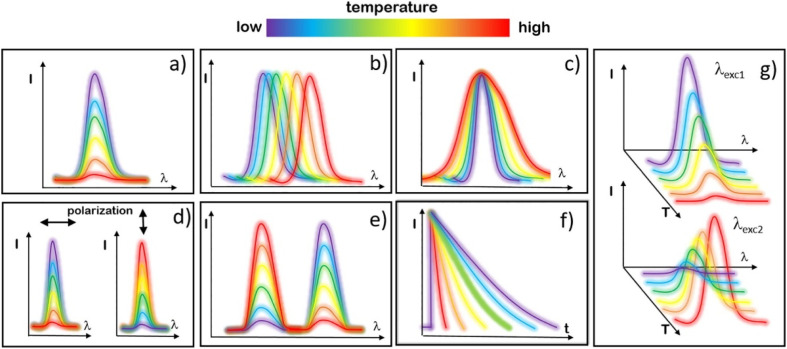
Various strategies for temperature readout in luminescence thermometry include (a) intensity-based (b) band-shift-based (c) bandwidth-based (d) polarization-based (e) ratiometric (f) kinetics-based and (g) single-band ratiometric. Reproduced with permission under Creative Commons CC BY 4.0 license from ref. 138 copyright@2022 The Authors.

Fig. 8(a)–(g) illustrates the classification of luminescence thermometry based on the specific luminescent parameters employed. Among the reviewed literature, the ratiometric approach emerges as the most widely adopted method.^138^ Consequently, this study primarily focuses on ratiometric temperature sensing, considering three key parameters: fluorescence intensity ratio (FIR), absolute sensitivity (*S*_A_), and relative sensitivity (*S*_R_), respectively.^139^ These parameters are defined as follows
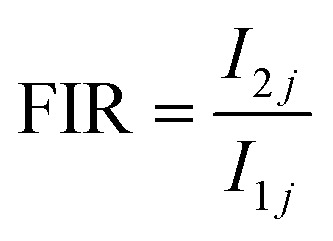

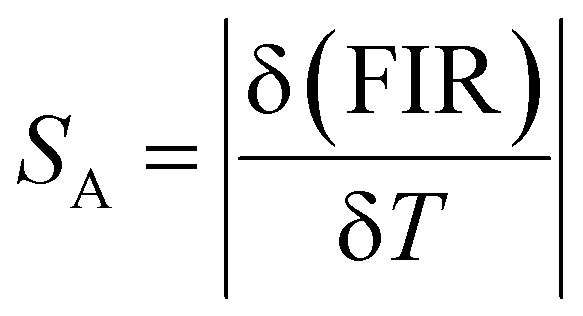

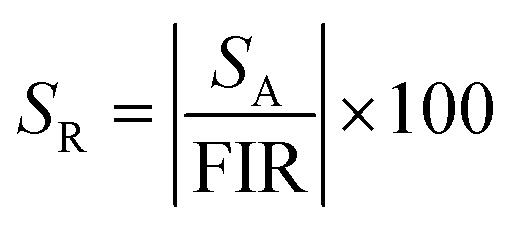
where *I*_*ij*_ is the fluorescence intensity for the transitions from the upper (*i* = 2) and lower (*i* = 1) thermalizing energy levels to a terminal level *j*, and *T* is the absolute temperature. Some of the recently reported temperature sensing properties of NPs include the work by Singh *et al.*, who focused on Ca_0.79−*x*_Bi_*x*_Er_0.01_Yb_0.2_MoO_4_ nano phosphors synthesized *via* gel-combustion methods.^140^ Here, co-doping with Bi^3+^ ions introduced local symmetry distortions around the Er^3+^ sites without altering the overall tetragonal CaMoO_4_ structure. This local asymmetry was found to significantly enhance the upconversion (UC) emission intensity by approximately 25 times compared to the undoped samples, primarily by suppressing non-radiative decay pathways. This enhancement resulted in an *S*_R_ value of 0.0068 K^−1^ at 300 K. However, this study was limited to room temperature measurements, and no absolute sensitivity value was presented, restricting its practical application scope for broader temperature ranges. Building upon this foundation, Gouraha *et al.* employed a microwave-assisted synthesis route to prepare Er_*x*_Yb_*y*_Ca_1−*x*−*y*_MoO_4_ nano phosphors.^141^ Microwave synthesis enabled finer control over particle size (∼15 nm), improved crystallinity, and rapid phase formation at lower energy inputs compared to conventional methods. The UC emission under 980 nm excitation was significantly improved, and temperature sensing capabilities were extended up to 398 K. The maximum *S*_R_ value reported was approximately 0.0170 K^−1^ at 398 K, indicating a better thermal response than that achieved by Singh *et al.* Moreover, Gouraha *et al.* expanded the functionality of the material by demonstrating catalytic efficiency in the selective oxidation of benzoin and benzyl alcohols, positioning these phosphors not only as optical sensors but also as potential catalysts. In a further progression, they also synthesized CaMoO_4_:Er^3+^/Yb^3+^ nanophosphors with enhanced upconversion and down conversion properties, again using a microwave-assisted approach but with optimized doping levels.^142^ The material exhibited robust temperature sensing capabilities, achieving a maximum *S*_A_ value of 10.74 × 10^−3^ K^−1^ at 500 K, thus significantly outperforming the previous two studies in terms of both sensitivity and operational temperature range. Despite these advancements, notable research gaps persist across all three studies. None of the studies addressed low-temperature sensing capabilities, leaving the behaviour of these materials below 300 K unexplored. Similarly, thermal cycling durability, which is essential for practical deployment in fluctuating temperature environments, remains unexplored. Next, we have presented the collective research on vanadate-based nanomaterials doped with various lanthanide ions (Tm^3+^, Er^3+^, Sm^3+^, Eu^3+^, Nd^3+^, Yb^3+^, and Dy^3+^), primarily focusing on significant advancements in morphology control, luminescent tuning, and temperature sensing applications. The study led Wang *et al.* synthesized LuVO_4_:Ln^3+^ (Ln = Tm, Er, Sm, Eu) nano/micro-structures *via* solvothermal synthesis using tartaric acid as a morphology-directing agent.^143^ They achieved the formation of diverse structures and demonstrated tunable multicolour emissions ranging from blue to white light by adjusting lanthanide dopant types and concentrations. However, while the study significantly advanced morphology-controlled photoluminescent materials, it lacked exploration into functional applications like thermal sensing, limiting its practical impact. Building upon structural achievements, the studies by Kolesnikov *et al.* introduced a crucial improvement by focusing on optical thermometry with LuVO4:Nd^3+^/Yb^3+^ nanophosphors.^144^ They exploited phonon-assisted UC mechanisms to achieve highly sensitive, non-contact temperature measurements. Improvements included achieving relative thermal sensitivities up to 2.6% K^−1^ and temperature resolutions as fine as 0.2 K across a wide temperature range of 323 to 873 K.^145^ These studies also analyzed the energy transfer efficiency between Nd^3+^ and Yb^3+^ centers and the influence of Yb^3+^ concentrations on thermometric performance aspects that were not addressed in Wang's earlier work. The 2023 paper, again by Kolesnikov *et al.* extended these advances to LaVO_4_ systems doped with Dy^3+^ and Sm^3+^. This study proposed a multimode thermometric approach, not just relying on luminescence intensity ratios, but also incorporating the temperature-induced red shift of the charge transfer band, a novel sensing mechanism enhancing sensitivity and reliability.^146^ The highest sensitivity achieved (*S*_r_ = 2.07% K^−1^ for LaVO_4_:Sm^3+^) for the temperature range 173–573 K. Nevertheless, this work was restricted to a moderate temperature range and focused on monoclinic LaVO_4_ rather than tetragonal LuVO_4_, hinting at a structural dependency that might limit generalizability. However, though Kolesnikov's works were functionally advanced, they still faced challenges related to long-term operational stability, potential agglomeration of NPs, and performance inconsistencies. Furthermore, scalability and repeatability under real-world conditions remain unexplored across all studies. Furthermore, the comparative analysis of YVO_4_-based luminescent nanomaterials doped with various lanthanide ions, across the studies conducted by Kolesnikov *et al.* (2017, 2018, 2021) and Hong Zhou (2020), shows a systematic evolution in optical nano thermometry applications. In 2017, Kolesnikov *et al.* synthesized Nd^3+^ doped YVO_4_ nanophosphors to function as non-contact thermal sensors based on near-infrared emission, specifically utilizing three independent thermometric parameters: FIR, spectral redshift (Stark level transitions), and full width at half maximum (FWHM) broadening.^147^ The study demonstrated that each parameter responded uniquely over a wide thermal window from 123 to 873 K, but the limited sensitivity of individual parameters and high thermal quenching at elevated temperatures exposed the need for more selective luminescent probes. Advancing this field, the 2018 study by Kalinichev under Kolesnikov's supervision introduced a ratiometric thermal sensing strategy utilizing the thermally coupled transitions between the ^4^F_5/2_ and ^4^F_3/2_ excited states of Nd^3+^ ions.^148^ By selecting a broader energy gap (several hundred cm^−1^) between these levels, the study achieved an increased Boltzmann population difference, leading to a markedly enhanced relative thermal sensitivity of up to 1.3% K^−1^, and allowed temperature detection with sub-degree resolution around 313 K and 673 K. Nevertheless, issues such as inhomogeneous particle sizes, aggregation tendencies in aqueous dispersions, and decreasing emission intensity at high dopant concentrations remained critical challenges. In 2020, Zhou *et al.* studied the temperature sensing properties of Pr^3+^ doped YVO_4_ phosphors by exploring both thermally coupled (^3^P_1_ → ^3^P_0_) and non-thermally coupled (^1^D_2_ → ^3^P_0_) level transitions.^149^ They demonstrated that the non-thermally coupled FIR technique, making use of transitions separated by a larger energy gap (∼1000 cm^−1^), could yield a higher *S*_R_ value of 1.137% K^−1^ at 313 K, outperforming conventional thermally coupled FIR methods. This improvement was attributed to reduced cross-relaxation pathways and minimized thermal back-transfer processes. However, the study noted slight distortions in the YVO_4_ crystal lattice after high-temperature sintering, potentially due to stress-induced anisotropic shrinkage, thus affecting luminescence stability under cyclic thermal stress. Finally, in the 2021 work led again Kolesnikov *et al.* systematically compared co-doped Eu^3+^/Nd^3+^ and singly doped Eu^3+^ and Nd^3+^ NPs.^150^ Co-doped systems exhibited moderate sensitivity (∼0.8% K^−1^) but suffered from non-radiative energy transfer inefficiencies, whereas the singly doped systems maintained discrete energy channels for each ion, resulting in enhanced thermal resolution down to 0.4 K. The use of ratiometric luminescence between the ^5^D_0_ → ^7^F_2_ transition of Eu^3+^ and the ^4^F_3/2_ → ^4^I_11/2_ transition of Nd^3+^, coupled with Boltzmann analysis, enabled precise and reproducible thermal readings, although slight inhomogeneities in nanoparticle dispersion introduced minor uncertainties in FIR calibration. But, in the case of Nd^3+^ based systems, the limited absorption cross-section at biological windows (∼800 nm) restricts application under low-power excitation; for Pr^3+^ and Eu^3+^ doped materials, concentration quenching and incomplete suppression of cross-relaxation mechanisms limit maximum achievable sensitivities.

#### 3.2.4 Limitations and challenges

Most of the existing studies have not thoroughly investigated critical parameters such as photobleaching resistance and long-term operational stability under continuous excitation factors that are essential for real-world biomedical and industrial applications. Collectively, the aforementioned studies, along with the findings summarized in Table 3, illustrate a clear progression from fundamental material characterization to application-driven developments in optical nano-thermometry. These advancements establish a solid foundation for future research aimed at the design of multifunctional, high-sensitivity luminescent temperature sensors tailored for photonic and industrial environments. Nevertheless, key challenges persist, particularly in enhancing operational durability, mitigating photobleaching, and optimizing energy transfer efficiency before such systems can achieve full commercial viability.

**Table 3:** List of recently reported nano phosphors for thermal sensing

Nano phosphor	Synthesis method	Temperature range (K)	Maximum absolute sensitivity	Ref.
BaTiO_3_:Er^3+^,Yb^3+^	Co-precipitation	300–505	0.00192 K^−1^ at 410 K	151
YMoO_4_:Er^3+^,Yb^3+^,Zn^2+^	Co-precipitation	300–523	0.0785 K^−1^ at 300 K	152
Lu_2_Ti_2_O_7_:Yb^3+^,Er^3+^	Hydrothermal	298–573	0.00313 K^−1^ at 536 K	153
CaSrSiO_4_:Tb^3+^	Sintering	10–290	17.6 × 10^−4^ K^−1^ at 70 K	154
NaZnPO_4_:Er^3+^,Eu^3+^,Yb^3+^	Co-precipitation	300–503	7.3 × 10^−3^ K^−1^ at 503 K	155
Gd_2_(MoO_4_)_3_:Er^3+^,Yb^3+^,Li^+^,Zn^2+^	Co-precipitation	300–473	38.7 × 10^−3^ K^−1^ at 473 K	156
Gd_2_O_3_:Er^3+^,Yb^3+^,Zn^2+^	Combustion	297–577	0.0116 K^−1^ at 297 K	157
NaGdF_4_:Yb^3+^,Ho^3+^,Ce^3+^	Hydrothermal	300–500	0.1446 K^−1^ at 500 K	158
NaGdF_4_: Yb^3+^,Ho^3+^	Hydrothermal	303–523	0.17 K^−1^ at 523 K	159
ZnGa_2_O_4_: Cr^3+^,Bi^3+^	Hydrothermal	303–503	0.017 K^−1^ at 493 K	160
LaOF:0.05Yb^3+^,Tm^3+^	Hydrothermal	303–573	0.0046 K^−1^ at 303 K	161

### 3.3 Thermoluminescence dosimetry applications

**Fig. 9:**
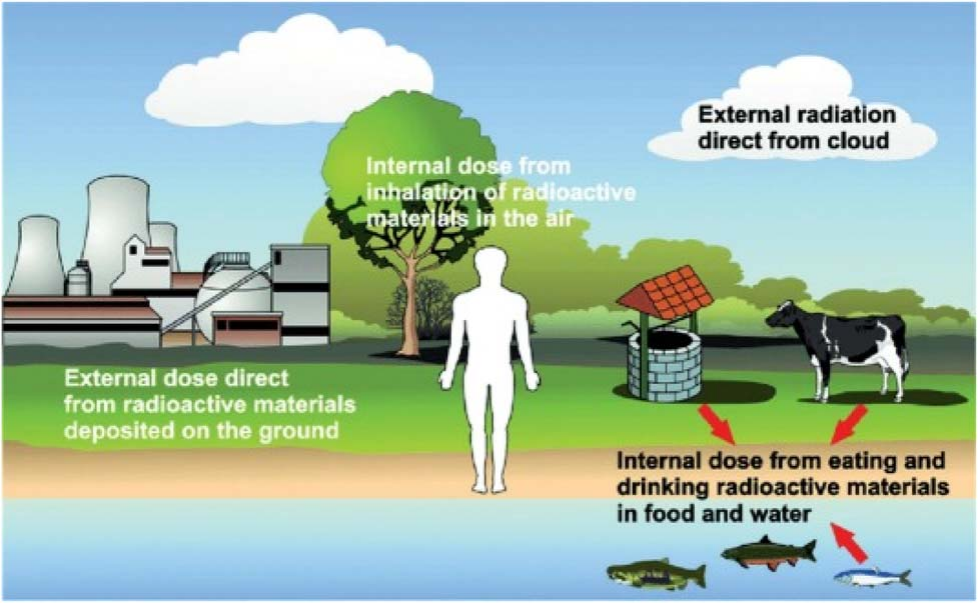
Various forms of artificial and natural radiation sources. Reprinted with permission from ref. 163, page no. 277, copyright (2022), Elsevier.

Humans are continuously exposed to different forms of radiation originating from various sources. As shown in Fig. 9, these sources can be broadly categorized into natural and artificial radiation sources.^162,163^ Among natural sources, the Sun is a major contributor of electromagnetic radiation. Other natural sources include terrestrial radionuclides, which are radioactive elements naturally present in the Earth's crust, and cosmogenic radiation, which results from interactions between cosmic rays and atmospheric particles.^164^ Environmental radiation is pervasive and is present in the soil, the food we consume, the water we drink, and the air we breathe. One of the most significant contributors to natural background radiation is radon gas, a radioactive decay product of uranium, which can be inhaled from the air or ingested through the food and water supply.^165^ On the other hand, artificial sources include technological devices such as mobile phones, as well as medical diagnostic procedures and industrial activities, which add to the overall radiation exposure, although typically at lower levels compared to natural sources.

The process of measuring and calculating the amount of radiation absorbed by a material is known as dosimetry. This is typically carried out using a small instrument known as a dosimeter.^166^ Among the various dosimetric methods available, the present study focused on the thermally stimulated luminescence (TSL) technique.^167^ The fundamental principle of this method is that the intensity of thermoluminescence (TL) emitted by a phosphor material is directly related to the radiation dose it has absorbed, thus allowing for the estimation of unknown radiation exposures. Thermoluminescent dosimeters (TLDs), which are widely used for this purpose, are manufactured in multiple forms, including powders, chips, rods, and ribbons.^168^ Their design also makes them convenient for personal use, commonly incorporated into wearable items such as badges and rings. The glow curve obtained during the TLD readout process provides critical information about the energy depth of the traps within the material that are responsible for the observed luminescence. Additionally, the linear response to varying radiation doses and the relatively low fading over time are key features that contribute to the widespread application of TLDs in radiation dosimetry.^169,170^

**Fig. 10:**
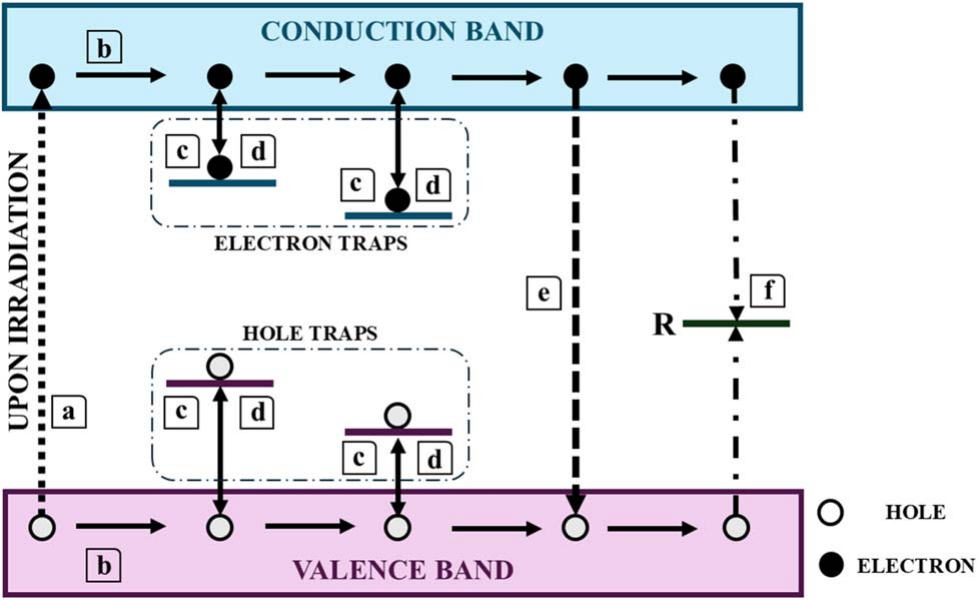
TL mechanism.

RENPs are integral to the study of TL, a process wherein light is emitted by wide-bandgap materials upon heating to sub-incandescent temperatures. This luminescent phenomenon arises not from the direct influence of heat but as a consequence of prior energy absorption from an external stimulus, with thermal energy functioning merely as a catalyst to release the stored energy. The usage of TL is particularly useful in dosimetry, as the intensity of emitted light correlates linearly with the absorbed energy. The intricate mechanisms underlying TL are illustrated in Fig. 10, delineated through sequential processes labeled as (a) to (e).^171^ The interaction of atoms with ionizing radiation generates free charge carriers (a), which travel through the crystalline lattice and become trapped at the trapping centers (b and c). Heating the TL material releases these carriers, leading to recombination (e and f), during which light is emitted if radiative recombination occurs.

Some of the released carriers may be retrapped (d) or recombined non-radiatively. A TL model includes traps and recombination centers, described by rate equations. The plot of thermoluminescence intensity against the heating rate is known as the TL glow curve. This curve is instrumental in determining key parameters, including activation energy (*E*), frequency factor (*s*), and order of kinetics (*b*), which characterize the TL glow peak. In the following section, we have compiled recent advancements in the field of TL dosimetry using RENPs.^171,172^ The field of radiation dosimetry has witnessed significant progress with the development of RENPs, particularly those activated with Eu^3+^ ions. From 2017 to 2024, a series of studies have explored various host matrices such as Y_2_O_3_, LiF, YVO_4_, BaSO_4_, ZnGa_2_O_4_, and SrMgAl_10_O_17_, incorporating Eu^3+^ and sometimes co-doped with other lanthanide ions like Sm^3+^ and Dy^3+^. These materials have been examined for their TL response, structural stability, and suitability for γ, β, X-ray, and proton dosimetry. One of the earliest foundational works in this timeline was conducted by Shivaramu *et al.*, who synthesized Eu^3+^ doped Y_2_O_3_ nanophosphors *via* the solution combustion method.^173^ Their study revealed that these materials demonstrated strong TL emission when exposed to γ-radiation, primarily due to the presence of deep electron trap centers. The TL glow curve was well defined, and the emission intensity correlated positively with dose, indicating suitability for dosimetric applications. The material's chemical stability and wide bandgap of 5.8 eV contributed to better glow curve reproducibility and minimal fading. Expanding on this, Kakade *et al.* synthesized Eu^3+^ doped Y_2_O_3_ using the sol–gel method.^174^ The study demonstrated that the structural and luminescent properties could be fine-tuned by adjusting Eu^3+^ concentration and annealing temperature. The TL studies revealed four distinct glow peaks at 100 °C, 117 °C, 139 °C, and 301 °C. While the first low-temperature peak faded rapidly, the others remained stable, offering a balance between sensitivity and signal retention. These characteristics established Y_2_O_3_:Eu^3+^ as a reliable material for high-dose TL applications, particularly in environmental and industrial monitoring. Kumar *et al.* focused on co-doped LiF nanophosphors containing Sm^3+^, Dy^3+^, and varying concentrations of Eu^3+^ ions.^175^ Synthesized *via* a chemical co-precipitation method, the LiF:Sm^3+^, Dy^3+^, and Eu^3+^ samples demonstrated excellent TL performance over an extended dose range of 0.1 to 30 kGy. The TL glow curve exhibited a strong peak between 398 K and 412 K. Using Chen's peak shape method and the TLAnal program, activation energies ranging from 0.71 to 2.24 eV were calculated along with frequency factors and kinetic orders. The phosphors showed linear TL behavior across the tested dose range and maintained a low fading of 10% over 27 days, making them highly effective for long-term radiation monitoring. This study also established the importance of deliberate defect engineering to enhance electron trapping and dosimetric response. Another study from the same group explored LiF co-doped with Sm^3+^ and Eu^3+^, revealing similar TL characteristics with a defined glow peak and consistent trap behaviour.^176^ These results suggested that co-doping not only improves TL intensity but also modifies trap depth, enabling a more stable dosimetric material. Next, Osorio *et al.* explored YVO_4_ doped with Eu^3+^ and co-doped with Dy^3+^ synthesized *via* the co-precipitation method.^177^ The inclusion of Dy^3+^ enhanced trap density and TL response by introducing deeper trap levels. The PL intensity and TL glow curves were influenced by crystallite size and preparation conditions. Importantly, the study found that these phosphors responded consistently to both β and γ irradiation, indicating their versatility in dosimetry. The ability of Dy^3+^ to act as a charge carrier trap significantly improved radiative recombination efficiency, leading to a higher and more stable TL output. Nattudurai *et al.* introduced BaSO_4_:Eu^3+^ as a novel phosphor for TL dosimetry under orthovoltage X-rays and low-energy proton irradiation.^178^ Using Co-60 γ-rays, the study demonstrated a linear TL response to doses ranging from 0.01 to 2 Gy. The maximum glow peak was recorded at 212 °C, with a TL signal that scaled linearly with absorbed dose across all radiation types. However, the material showed strong energy dependence, with TL calibration slopes varying significantly between X-ray and proton beams. Despite this, the material's reusability, small size, and high sensitivity made it a valuable dosimeter if properly calibrated in the user beam. A more recent investigation by Barad *et al.* involved ZnGa_2_O_4_:Eu^3+^ synthesized using gel combustion.^179^ The TL glow curve analysis identified five distinct peaks, with the main peak around 190 °C. The dose–response curve showed excellent linearity up to 100 Gy with *R*^2^ = 0.989 and a sublinear response beyond that range. Deconvolution techniques were used to determine trap depth and frequency factors. The optimal dopant concentration was found to be 2% Eu^3+^. The high chemical and thermal stability of the ZnGa_2_O_4_ host lattice, along with strong persistent luminescence and a high density of trap centers, makes it a suitable candidate for medium-dose range dosimetry, especially in industrial or medical contexts. While each system showed specific advantages, some recurring limitations were observed. In materials like BaSO_4_, energy dependence under different radiation sources can limit universal application unless specific calibrations are performed. Low-temperature glow peaks, especially in Y_2_O_3_ and LiF systems, were prone to fading due to shallow trap levels, affecting long-term signal reliability. Additionally, synthesis techniques such as gel combustion and sol–gel methods, while offering fine control over morphology and dopant distribution, may pose challenges in scalability and reproducibility across larger production batches. Nonetheless, significant improvements have been made in the field. The evolution from singly doped to co-doped systems enhanced both TL intensity and trap complexity. Co-dopants like Dy^3+^ and Sm^3+^ proved effective in introducing deeper and more stable traps, broadening the dose–response range, and reducing signal fading. In conclusion, recently a considerable advancement in the design and application of Eu^3+^ activated nanophosphors for TL dosimetry. In the next few papers, we have provided the evaluation of five similar host systems doped with Dy^3+^. We start with the study by Bahl *et al.*, who synthesized a novel co-doped phosphor CaSO_4_:Dy,Mn using a modified recrystallization method.^180^ Their primary goal was to enhance the sensitivity of the well-established CaSO_4_:Dy phosphor, which, despite its wide usage in medical and environmental dosimetry, required improved performance for lower dose detection. The introduction of Mn as a co-dopant at an optimized ratio of Dy : Mn = 0.025 : 0.075 within a fixed total concentration of 0.1 mol% significantly increased the TL intensity, almost doubling that of the conventional CaSO_4_:Dy and surpassing that of LiF:Mg,Cu,P. Importantly, the TL glow peak remained at ∼240 °C, a desirable temperature for practical dosimetry applications, and the phosphor displayed a stable fading profile, with only ∼11% signal loss over three months. The glow curve deconvolution revealed a complex trapping mechanism, yet it retained reproducibility and linearity in the low-dose range, making it especially suitable for applications such as diagnostic radiology and nuclear medicine, where precise detection of microgray level exposures is crucial. Complementing this development, Mandlik *et al.* focused on extending the dosimetric range of CaSO_4_:Dy by reducing its particle size to the nanometer regime through a chemical co-precipitation technique.^181^ The resulting nanorod-shaped CaSO_4_:Dy, with dimensions around 20 nm in diameter and 200 nm in length, exhibited a major TL glow peak at approximately 283 °C after annealing at 700 °C. Structural analyses *via* XRD and TEM confirmed phase changes and morphological evolution during annealing, which directly influenced the TL behaviour. This nanophosphor demonstrated a wide and unsaturated dose response up to 10 kGy, unlike its microcrystalline counterpart, which is saturated at ∼100 Gy. Moreover, fading studies showed only minor signal degradation, reinforcing its potential for high-dose dosimetry applications such as sterilization, industrial irradiation, and radiation therapy monitoring. In a related study, Mandlik *et al.* investigated the CaSO_4_:Eu nanophosphors, emphasizing the impact of particle size on both TL and PL properties.^182^ The microcrystalline phosphor was first prepared *via* the acid recrystallization method and subsequently subjected to ball milling to yield various particle sizes, ranging from 5 μm to as small as 50 nm. Additionally, nanocrystalline CaSO_4_:Eu was synthesized using the chemical co-precipitation method for comparison. It was observed that the TL intensity of the phosphor decreased significantly with particle size reduction, particularly for samples under 200 nm. This trend was attributed to the increased presence of surface defects and non-radiative recombination centers, which quench the luminescence. Despite this, the 50 nm sample maintained a linear TL response over a wide dose range of 1–10 kGy, making it suitable for both low and high radiation dose applications. The study also confirmed that Eu ions were present in both Eu^2+^ and Eu^3+^ states, contributing to the dual-mode emission observed in PL spectra. While NPs offer enhanced surface area and tunable properties, it is critical to balance grain size and structural integrity to preserve luminescence performance. Similarly, the TL behaviour of sodium sulfate-based phosphors was explored by Vidya *et al.* through the synthesis of Na_2_SO_4_:Dy and LiNaSO_4_:Dy *via* the slow evaporation technique, followed by calcination at 400 °C.^183^ Their work revealed that Dy^3+^ incorporation into Na_2_SO_4_ led to the appearance of glow peaks around 61 °C and 175 °C, indicating the formation of shallow and deep traps, respectively. Meanwhile, the co-doping with Li^+^ ions in LiNaSO_4_ resulted in a significant shift in glow peak positions and a general reduction in TL intensity. The authors interpreted this behaviour as the result of lithium-induced structural changes and possible recombination suppression due to charge compensation mechanisms. Despite these effects, both phosphors exhibited linear dose responses up to 5 kGy and good reproducibility, suggesting their utility for mid-dose dosimetry applications. Additionally, the activation energies and frequency factors were derived using peak shape analysis, offering deeper insight into the trap dynamics of these systems. However, the relatively low glow peak temperatures may limit their use in thermally unstable environments or field deployments with fluctuating ambient conditions. The most recent and advanced material in this comparative review is the Dy^3+^ doped Zn_0.66_Mg_0.3_Al_2_O_4_ phosphor, synthesized by Pathak *et al.* employing a solution combustion synthesis route, The researchers prepared a spinel-phase nanophosphor optimized with 4 mol% Dy^3+^ ions.^184^ Structural characterization *via* XRD, FTIR, and SEM confirmed the successful incorporation of Dy^3+^ into the host lattice and the presence of nanostructured particles (∼10–25 nm). TL measurements revealed glow peaks at 246–250 °C across doses ranging from 600 to 1000 Gy, with nearly linear dose–response behavior and minimal fading of nearly 4.25% over 20 days. The high activation energy, ranging from 0.93 to 0.97 eV, and deep trap characteristics suggest excellent stability and low thermal noise, rendering the material highly suitable for high-dose applications such as nuclear reactor surveillance, space mission dosimetry, and industrial accelerator operations. Moreover, a substantial body of literature has been dedicated to exploring the dosimetric applications of NPs, with numerous pioneering studies summarized in Table 4.

**Table 4:** Recently reported NPs and their TL properties

Nano phosphor	Radiation type	Dose	Deconvoluted TL peaks (K)	Order of kinetics	*E* (eV)	Frequency factor (s^−1^)	Ref.
Lu_3_Al_5_GaO_12_:Ce^3+^	β rays	50 Gy	323.3	1	0.65	2.51 × 10^11^	187
			343.1	1	0.68	2.37 × 10^11^	
			361	1	0.72	2.25 × 10^11^	
			388.4	1	0.78	2.09 × 10^11^	
ZnAl_2_O_4_:Dy^3+^	γ rays	10 Gy	446	1	0.17	1.9 × 10^5^	188
			469	1	0.11	1.7 × 10^5^	
			598	1	0.37	1.0 × 10^5^	
			648	1	0.59	2.7 × 10^5^	
SrZr_4_(PO_4_)_6_:Pr^3+^	UV	30 min	486	2	0.69	1.64 × 10^6^	189
MgO:Li,Sm	γ rays	15 Gy	442	1.9	1.37	1.8 × 10^15^	190
CdSiO_3_:Gd^3+^	UV	10 min	346.25	2	0.68	2.62 × 10^8^	191
			392.05	2	0.67	7.61 × 10^6^	
			436.76	2	0.95	7.59 × 10^8^	
KCl:Sm^3+^	Proton ion	250 keV	535	1	1.51	2.6 × 10^13^	192
			583	2	1.65	1.9 × 10^14^	
			512	1	1.31	8.5 × 10^11^	
	Carbon ion	250 keV	550	1	1.10	7.2 × 10^10^	
			606	2	1.45	6.2 × 10^12^	
			608	1	0.99	5.9 × 10^9^	
K_2_Ca_2_(SO_4_)_3_:Eu^3+^	Electrons	6 MeV	423	1	0.97	1.29 × 10^11^	193
			439	1	1.08	8.50 × 10^11^	
			477	1	1.23	3.21 × 10^12^	
			532	1	0.74	1.81 × 10^6^	
	γ rays	10 kGy	422	1	0.99	2.66 × 10^11^	
			435	1	1.03	2.73 × 10^11^	
			479	1	1.24	3.88 × 10^12^	
			532	1	0.75	1.72 × 10^6^	
LiMgBO_3_:Dy^3+^	Ag^9+^	120 MeV	355	2	0.92	4.22 × 10^12^	194
			387	2	0.72	6.28 × 10^8^	
			410	2	0.65	2.45 × 10^7^	
			567	2	0.88	1.11 × 10^7^	
	γ rays	5 Gy	376	2	1.26	3.65 × 10^16^	
			397	2	1.07	1.79 × 10^13^	
SnO_2_:Eu^3+^	γ rays	15 kGy	366.5	1.40	0.65	2.36 × 10^8^	195
			440	1.56	0.98	4.73 × 10^10^	
			529	1.42	1.10	6.46 × 10^9^	
			624	2.00	1.17	2.58 × 10^10^	
CaF_2_:Ce^3+^	γ rays	5 Gy	394	2.02	1.73	3.2 × 10^21^	196
			411	1.63	1.24	2.8 × 10^14^	
			425	1.66	1.65	9.4 × 10^18^	
			445	1.73	1.17	2.1 × 10^12^	
			556	1.30	1.97	1.1 × 10^17^	
			594	1.57	1.84	4.9 × 10^15^	
			632	1.14	1.49	6.4 × 10^10^	

#### 3.3.1 Limitations and challenges

These investigations collectively contribute to a growing knowledge base on TL phosphor development, providing both theoretical and practical frameworks for customizing materials to meet diverse dosimetric requirements. Currently, the development of novel phosphor materials largely relies on empirical trial-and-error methods. A primary challenge is to establish a systematic design approach that enables the prediction of phosphor behavior based on targeted properties. Computational tools, particularly density functional theory (DFT), offer significant potential in predicting key parameters such as trap types, energy levels, and optimal host-dopant combinations. A second challenge involves the rational engineering of trap centers. Effective phosphor performance requires deep trap centers for enhanced charge storage, while shallow traps contribute to latent image stability. Additionally, minimizing spatial overlap between traps is crucial to avoid cross-talk. Selecting dopants with narrow and discrete trap energy distributions is also essential.^185^ Future research must focus on the accelerated development of next-generation dosimeter technologies, with an emphasis on benchmarking their performance against existing commercial systems. Also, it is necessary to integrate co-doping, nanostructuring, and host lattice engineering into multifunctional composite materials capable of spanning wider dose ranges with minimal fading, high thermal stability, and consistent linearity. Although challenges like energy dependence and synthesis complexity remain, the growing body of knowledge and continual optimization of trap structures, emission properties, and material stability pave the way for the next generation of smart, high-performance radiation dosimeters.^186^

## 4. Conclusions

In conclusion, this paper highlights the significant advancements in the synthesis and optimization of rare-earth-doped nano phosphors (RENPs), with a focus on the strategic selection of host materials such as tungstates, vanadates, and aluminates. The incorporation of rare-earth elements has proven to significantly enhance the PL properties and efficiency of these materials, leading to the development of phosphors with tunable emission spectra, high luminous efficiency, and desirable chromaticity for advanced lighting applications. The careful modulation of dopant concentrations and energy transfer mechanisms plays a critical role in optimizing these materials for specific applications, particularly for lighting technologies. Furthermore, the UCNPs have been demonstrated for various advanced sensing applications, alongside electrochemical and photocatalytic applications. Also, the use of synthesis methods such as sol–gel, combustion, and microwave-assisted techniques further improves their performance for environmental and biomedical monitoring. Lastly, RENPs exhibit exceptional promise in TL dosimetry, showcasing their ability to provide precise radiation dose measurements with minimal fading and reliable dose-dependent responses, which underscores their suitability for medical and environmental dosimetry applications. These findings open the door for the further development and implementation of RENPs in a broad range of scientific and industrial fields.

## Conflicts of interest

The authors declare that they have no known competing financial interests or personal relationships that could have appeared to influence the work reported in this paper.

## Data Availability

This review does not include any primary research findings, software, or code, nor does it present new data generation or analysis.
